# Branched-chain amino acid catabolism is potentially deficient in pancreatic cancer with high-neural invasion

**DOI:** 10.1038/s41598-026-47741-x

**Published:** 2026-04-12

**Authors:** Hiroki Michida, Asami Hagiwara, Yuki Saito, Noriko Kawasaki, Mayuka Kanda, Mika Kawasaki, Sachise Karakawa, Hidetaka Suzuki, Masafumi Ikeda, Hideki Makinoshima, Shuichi Mitsunaga

**Affiliations:** 1 Research Institute for Bioscience Products and Fine Chemicals, Ajinomoto Co., Inc., Kawasaki, Japan; 2 Division of Biomarker Discovery, Exploratory Oncology Research & Clinical Trial Center, National Cancer Center, Kashiwa, Japan; 3 Department of Pharmacy, National Cancer Center Hospital East, Kashiwa, Japan; 4 Department of Hepatobiliary and Pancreatic Oncology, National Cancer Center Hospital East, Kashiwa, Japan; 5 Tsuruoka Metabolomics Laboratory, National Cancer Center, Tsuruoka, Japan; 6 Division of Translational Informatics, Exploratory Oncology Research and Clinical Trial Center, National Cancer Center, Kashiwa, Japan; 7 Division of Biomarker Discovery, Exploratory Oncology Research & Clinical Trial Center, National Cancer Center, Kashiwanoha 6-5-1, Kashiwa, Chiba 277-8577 Japan

**Keywords:** Branched-chain amino acid, Branched-chain α keto acid dehydrogenase, Pancreatic cancer, Nerve invasion, Cancer, Diseases, Endocrinology, Gastroenterology

## Abstract

Elevated plasma branched-chain amino acid (BCAA) levels are observed in some metabolic disorders, such as obesity and diabetes, due to impaired hepatic BCAA catabolism. In pancreatic cancer, elevated plasma BCAA levels are observed years before diagnosis, possibly due to increased protein degradation. However, the status of BCAA catabolism in pancreatic cancer remains unclear. Here, we hypothesized that defective hepatic BCAA utilization contributes to this elevation in pancreatic cancer, similar to observations in obesity and diabetes. Consistent with our hypothesis, increased phosphorylation of the hepatic BCAA catabolic enzyme, branched-chain α-keto acid dehydrogenase (BCKDH), was observed in a murine nerve invasion model with human pancreatic cancer cells, indicative of an inactive state. Notably, this was associated with reduced whole-body BCAA flux, increased skeletal muscle degradation, and elevated plasma BCAA levels. Patients with stage III pancreatic cancer with severe neural invasion exhibited high plasma BCAA and reduced muscle mass. In addition, patients with stage IV pancreatic cancer showed hepatic BCKDH phosphorylation, which correlated with poor prognosis. Overall, these results suggest the potential existence of impaired BCAA catabolism via hepatic BCKDH downregulation in pancreatic cancer.

**Supplementary Information:**

The online version contains supplementary material available at 10.1038/s41598-026-47741-x.

## Introduction

Branched-chain amino acids (BCAAs), which consist of valine, leucine, and isoleucine, are essential amino acids not synthesized by humans^1^. The internal BCAA pool in the body is controlled by the balance among the food supply (mainly protein), digestion, protein breakdown or biosynthesis mediated by gut bacteria, and consumption for protein synthesis or energy generation^2^. When BCAAs are catabolized for energy generation, they first undergo reversible transamination catalysis to generate their corresponding branched-chain keto acids (BCKAs; consisting of α-ketoisovalerate, α-KIV; α-ketoisocaproate, α-KIC; and α-keto-β-methylvalerate, α-KMV), mediated by BCAA transaminase (BCAT), mainly in peripheral tissues, such as the muscles^1^ and white adipose tissues^3^, owing to the limited expression of BCAT in the liver^1^. Then, BCKAs are transferred to the liver via the circulation and decarboxylated by branched-chain α-keto acid dehydrogenase (BCKDH), a rate-limiting enzyme involved in BCAA catabolism^4^. BCKDH consists of three subunits (E1, E2, and E3) and is regulated by reversible phosphorylation of the E1 subunit at Ser^293^^5^. Under fasting conditions, the activity of BCKDH is increased^6^, and this is regulated by the phosphorylation of the E1 subunit at Ser^293^^5^.

BCAA levels in plasma are altered in various disorders. Epidemiological studies have indicated that elevated plasma BCAA levels are associated with insulin resistance in obesity^7^ and coronary artery disease^8^. In contrast, decreased plasma BCAA levels are associated with chronic renal and liver failure^9,10^. Previous studies have attributed elevated plasma BCAA levels in these disorders to impaired BCAA catabolism. For instance, diabetes and obesity are characterized by increased phosphorylation of BCKDH E1 Ser^293^ (deactivated state of BCKDH) and decreased whole-body BCAA flux^11,12^, resulting in elevated plasma BCAA levels.

Importantly, altered plasma BCAA levels have been reported in various cancers, including leukemia and breast cancer^13,14^. In pancreatic cancer, elevated plasma BCAA levels due to muscle loss has been observed in *LSL–Kras*^*G12D/*+^*;LSL–Trp53*^*R172H/*+^*;Pdx–1–Cre* (KPC) murine model expressing mutant Kirsten rat sarcoma virus oncogene homologue (KRAS), using isotope-labeled amino acid diets^15^. However, whether impairment in BCKDH activity in the liver contributes to elevated plasma BCAA levels in diseases such as obesity and diabetes remains unclear^11,12^.

In a previous study, we established a murine nerve invasion model harboring human pancreatic cancer cells (N-inv model), which showed whole-body metabolic changes triggered by glial activation in the central nervous system^16^. Neural invasion is a common feature of human pancreatic cancer and is associated with poor prognosis^17,18^. In this study, we aimed to investigate the existence of defective hepatic BCAA utilization in pancreatic cancer using the N-inv model, which showed elevated plasma BCAA levels during fasting. In particular, we performed whole-body leucine flux analysis and molecular analysis of BCAA catabolic enzymes. In addition, we assessed the translation and clinical relevance of the findings from the N-inv model in patients with pancreatic cancer.

## Results

**Fig. 1:**
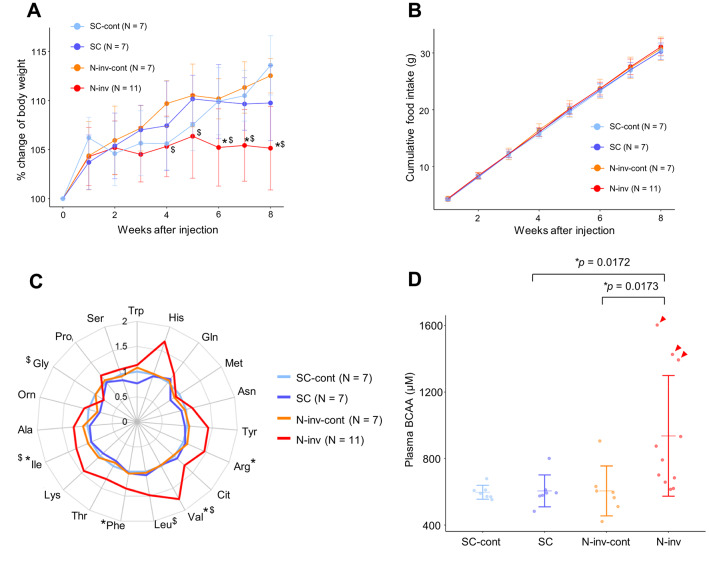
Phenotypes of murine models after injection of human pancreatic cancer cells.(**A**) Percentage change in body weight and (**B**) cumulative food intake after Capan-1 or PBS injection. (**C**) Plasma amino acid profile in murine models at 8 weeks after cell injection. Data are normalized to the SC-cont group. (**D**) Total plasma BCAA levels in each murine model at 8 weeks after cell injection. All data in this figure are from mice in cohort 1. Arrowheads indicate extremely high N-inv values. “*” and “$” in (**A**), (**C**), and (**D**) indicate significant differences in N-inv vs. SC and N-inv vs. N-inv-cont comparisons, respectively at *p* < 0.05 (one-way ANOVA with Dunnett’s post-hoc test). Representative data in (**A**), (**B**), and (**D**) are shown as mean ± standard deviation. SC, subcutaneous tumor model; N-inv, neural invasion model; SC-cont, sham control group for SC; N-inv-cont, sham control group for N-inv; Trp, tryptophan; His, histidine; Gln, glutamine; Met, methionine; Asn, asparagine; Tyr, tyrosine; Arg, arginine; Cit, citrulline; Val, valine; Leu, leucine; Phe, phenylalanine; Thr, threonine; Lys, lysine; Ile, isoleucine; Ala, alanine; Orn, ornithine; Gly, glycine; Pro, proline; Ser, serine; BCAA, branched-chain amino acid.

Phenotypes of murine models Two mouse model cohorts (animal cohorts 1 and 2, see Methods) were generated in this study. We first assessed the phenotypes of murine models using animal cohort 1. In particular, we generated subcutaneous tumor (SC) and neural invasion (N-inv) models by injecting the Capan-1 human pancreatic cancer cell line into the subcutaneous tissue of the left flank or left sciatic nerve of severe combined immunodeficiency (SCID) mice. In addition, we created sham control groups for the SC and N-inv models by injecting phosphate-buffered saline (PBS) into the same site in each tumor model (SC-cont and N-inv-cont). Importantly, the growth of mice in the N-inv group stopped 2 weeks after cell injection, and their body weights were significantly lower than those of mice in the N-inv-cont and SC-group mice after 4 and 6 weeks, respectively (Fig. 1A). Supplementary Fig. 1 shows the changes in the weight of mice in the cohort 2. No significant differences were observed in cumulative food intake between the N-inv and SC or N-inv-cont groups during the study period (Fig. 1B). In addition, the difference in body weight between the N-inv and SC models was conserved after overnight fasting (Supplementary Table 1) and was mostly attributed to the weights of the liver and white adipose tissue (Supplementary Table 1). Tumor weights were comparable between the N-inv and SC groups (Supplementary Table 1). Moreover, the mRNA expression of glial fibrillary acidic protein 1 (*Gfap1*) in the spinal cord, a molecular marker of neuroinflammation, was significantly higher in the N-inv group than in the SC group, as previously reported^16^ (Supplementary Table 1). In addition, we examined whole body and tissues weight and *Gfap1* expression in the spinal cord of animals in cohort 2 and confirmed the reproducibility of these characteristics in the N-inv group (Supplementary Table 2).

Furthermore, we quantified plasma amino acid levels in each model under fasting conditions in cohort 1. BCAA (valine and isoleucine), phenylalanine, and arginine levels were significantly higher in the N-inv group than in the SC group (Fig. 1C). Similarly, BCAA (valine, isoleucine and leucine) levels were significantly higher in the N-inv group than in the control (N-inv-cont) group. In contrast, glycine level was significantly lower in the N-inv group than in the N-inv-cont group. In addition, the total BCAA level was significantly higher in the N-inv group than in the SC and N-inv-cont groups (Fig. 1D). Importantly, several mice in the N-inv group exhibited extremely high plasma BCAA levels, suggesting disrupted BCAA metabolism. To investigate the metabolic features of these N-inv-model mice with extremely high plasma BCAA levels, we stratified N-inv mice into two groups based on plasma BCAA levels using the k-means clustering method. Accordingly, three N-inv-group mice with extremely high plasma BCAA levels (Supplementary Fig. 2 A) and defined as “*type severe*,” whereas the other N-inv-group mice classified as “*type moderate*.” Although the number of *type severe* mice was only three, we confirmed the reproducibility of N-inv *type severe* mice in cohort 2 (Supplementary Fig. 2B).

### Status of hepatic BCKDH and whole-body leucine flux in murine models

**Fig. 2:**
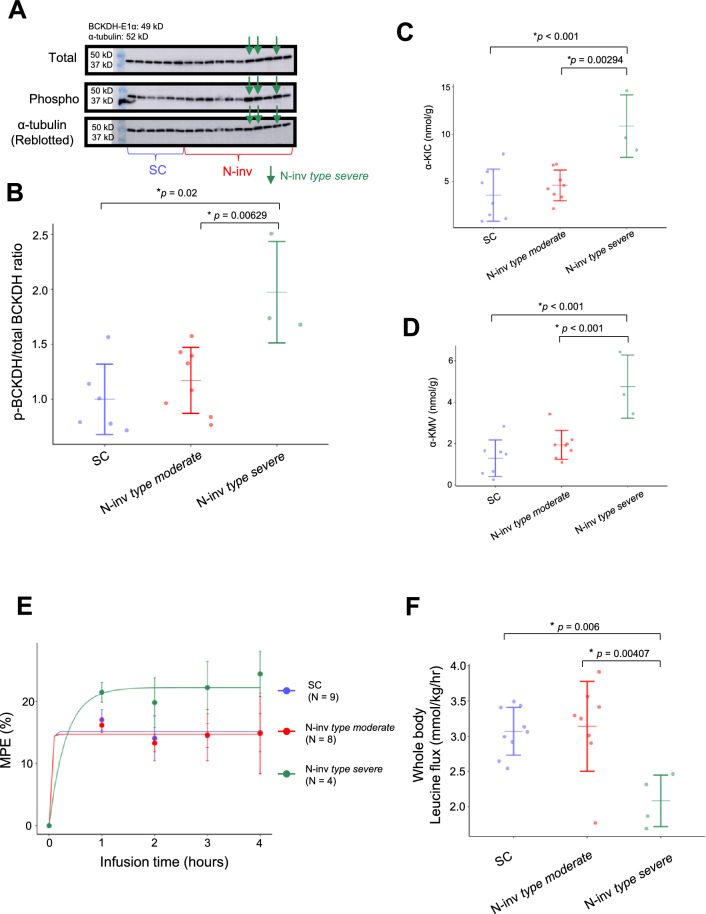
Analysis of whole-body leucine flux and BCAA catabolism status in murine models. (**A**) Western blot images of total liver BCKDH, phosphorylated BCKDH, and α-tubulin. The image of α-tubulin is a re-blot. Arrows indicate the bands for N-inv *type severe* group. The blots were cropped, and the original blots are presented in Supplementary Fig. 6 A with labels and Supplementary Fig. 7 A without labels. (**B**) Hepatic BCKDH phosphorylation levels in murine models. Phosphorylated BCKDH levels were normalized to those of total BCKDH. In addition, the values were normalized to the average value of the SC group. Concentrations of (**C**) α-KIC and (**D**) α-KMV in the livers of murine models. (**E**) Averaged MPE of ^15^N-leucine at each time point based on the infusion test using each murine model. Solid line: fitted mathematical model. Bars: standard deviation. For details, see the Materials and Methods section. (**F**) Whole body leucine flux in murine models. Representative data are shown as the mean ± standard deviation in **B**–**D** and F. *p < 0.05, based on Dunnett’s post hoc test. (**A**–**D**) are the data from animal cohort 1, and (**E**–**F**) were obtained from animal cohort 2. MPE, mole percent excess; SC, subcutaneous tumor model; N-inv, neural invasion model; SC-cont, sham control group for SC; N-inv-cont, sham control group for N-inv; BCKDH, branched-chain α-keto acid dehydrogenase; Phospho, phosphorylated; p-BCKDH, phosphorylated BCKDH; α-KIC, α-ketoisocaproate; α-KMV, α-keto-β-methylvalerate.

To evaluate BCAA utilization in the liver in the N-inv *type severe* group, we evaluated the phosphorylation of the E1 subunit Ser^293^ of hepatic BCKDH using liver samples from cohort 1. Notably, the phosphorylation level increased significantly in the N-inv *type severe* group (2.0 ± 0.46) compared with that in the SC group (1.0 ± 0.32, *p* = 0.02) and N-inv *type moderate* group (1.2 ± 0.30, *p* = 0.00629) (Fig. 2A,B). In addition, we measured the concentrations of BCKAs, specifically α-KIC and α-KMV, in liver tissue. Importantly, α-KIC and α-KMV levels were significantly higher in the N-inv *type severe* group (11 ± 3.3 and 4.8 ± 1.5 nmol/g, respectively) than in the SC group (3.6 ± 2.7 and 1.3 ± 0.88 nmol/g, respectively; *p* < 0.001) and N-inv *type moderate* group (4.6 ± 1.6 nmol/g [*p* = 0.00294] and 1.9 ± 0.70 nmol/g [p < 0.001], respectively) (Fig. 2C,D).

Furthermore, we examined the mRNA expression levels of branched-chain amino acid transaminase 2 (*Bcat2*), an enzyme involved in BCAA catabolism, in samples from animal cohort 1. *Bcat2* mRNA expression in white adipose tissue (but not in skeletal muscle and liver) was significantly lower (*p* = 0.0239) in the N-inv *type severe* group than in the N-inv *type moderate* group (Supplementary Fig. 4).

Moreover, we measured BCAA metabolic flux to investigate the effects of increased hepatic BCKDH phosphorylation on the whole-body metabolic capacity of BCAA, using cohort 2. BCAA flux analysis was conducted using a stable isotope-labeled ^15^N-leucine infusion experiment in a fasting state. When isotope-labeled leucine is infused into a mouse at a constant rate, the isotopic enrichment of leucine in the plasma increases until it reaches a plateau, at which time the metabolic steady state is achieved. Given that the isotopic enrichment of leucine is inversely related to the rate at which internal leucine appears and disappears in the plasma of mice, this experiment allowed us to quantify the whole-body leucine flux in mice. Figure 2E shows that the mole percent excess (MPE), a parameter of isotopic enrichment, stably plateaued at different values in each model in our experiments. Whole-body leucine flux was significantly lower in the N-inv *type severe* group (2.1 ± 0.36 mmol/kg/h) than in the SC (3.1 ± 0.34 mmol/kg/h, *p* = 0.006) and N-inv *type moderate* (3.1 ± 0.64 mmol/kg/h, *p* = 0.00407) groups (Fig. 2F).

### Metabolic characterization of N-inv *type severe* group

**Fig. 3:**
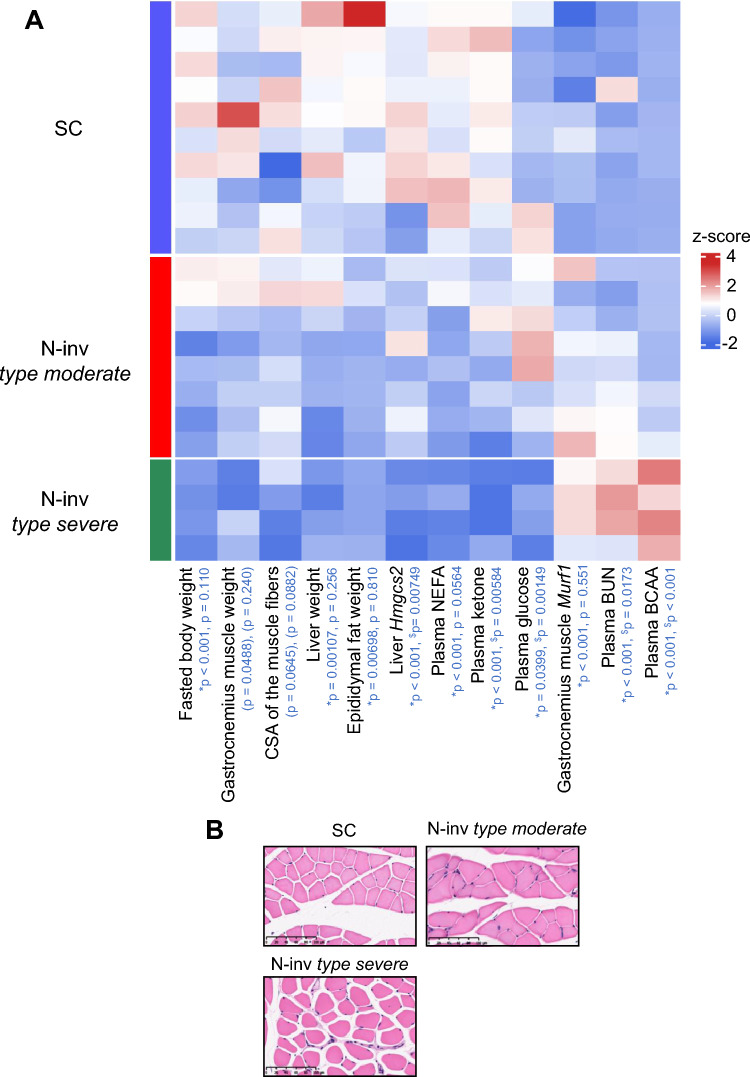
Metabolic characterization of murine models. (**A**) Heatmap of metabolic indices in each mouse in cohort 2. Only the right gastrocnemius muscle was weighed because the Capan-1 human pancreatic cancer cell line or PBS was injected into the left sciatic nerve or the subcutaneous tissue of the left flank of mice. Epididymal fat was weighed bilaterally. Each row corresponds to each mouse. NEFA and BUN were measured using plasma from blood samples collected from the vena cava at the end of the infusion test. Ketone, glucose and total BCAA were measured using plasma from blood samples collected from the tail vein immediately before the infusion test. The color indicates the z-score. Two *p*-values based on Dunnett’s post hoc test, for the comparisons of N-inv *type severe* vs. SC and N-inv *type severe* vs. N-inv *type moderate*, respectively, are shown in blue letters. * and $ indicate significant differences in N-inv *type severe* vs. SC and N-inv *type severe* vs. N-inv *type moderate* comparisons at *p* < 0.05. The *p*-values calculated based on Dunnett’s post hoc test for comparisons that were not significant in the one-way ANOVA are shown in parentheses. (**B**) Representative stained image of gastrocnemius muscle fibers in mice in cohort 2. Scale bar = 100 μm. SC, subcutaneous tumor model; N-inv, neural invasion model; CSA of the muscle fibers, cross-sectional area of the gastrocnemius muscle fibers; *Hmgcs2*, mRNA expression level of 3-hydroxy-3-methylglutaryl-CoA synthase 2; NEFA, non-esterified fatty acid; *Murf1*, mRNA expression level of muscle ring finger protein 1; BUN, blood urea nitrogen; BCAA, branched-chain amino acid.

We investigated the metabolic features of mouse models in cohort 2 by evaluating liver weight (proxy of hepatic glycogen or lipid content), ketogenesis-related indices (white adipose tissue weight, plasma non-esterified fatty acid [NEFA] levels, plasma ketone levels, and hepatic 3-hydroxy-3-methylglutaryl-CoA synthase 2 [*Hmgcs2*] mRNA expression), plasma glucose level (proxy of gluconeogenesis), and protein catabolism markers (gastrocnemius muscle weight, cross-sectional area of the gastrocnemius muscle fibers, plasma blood urea nitrogen [BUN], and muscle ring finger protein 1 (*Murf1*) mRNA expression in gastrocnemius muscle). N-inv *type severe* mice were characterized by lower gastrocnemius muscle weight, smaller gastrocnemius muscle fibers, higher plasma BUN levels, upregulated *Murf1* expression in the gastrocnemius muscle, lower plasma NEFA and ketone levels, downregulated hepatic *Hmgcs2* expression, lower adipose tissue weight, and reduced plasma glucose levels (Fig. 3A). Collectively, these results indicate increased protein breakdown and decreased fat oxidation and gluconeogenesis, suggesting a state of severe energy depletion. Representative stained image of gastrocnemius muscle fiber is shown Fig. 3B. Importantly, we confirmed that the characteristics of N-inv *type severe* mice were consistent with findings in cohort 1 (Supplementary Fig. 2 C). In addition, we examined the plasma amino acid levels of mice in cohorts 1 and 2 (Supplementary Fig. 3 A, B). Notably, the plasma levels of various amino acids, particularly BCAAs, were upregulated in the N-inv *type severe* mice, indicating increased protein catabolism.

Overall, elevated plasma BCAA levels, enhanced protein catabolism, and reduced fat oxidation and gluconeogenesis were identified as metabolic features of the fasting N-inv *type severe* group.

### Metabolic characterization of patients with pancreatic cancer

To examine the physiological relevance of the findings in the N-inv model, we investigated the metabolic features of patients with stage III pancreatic cancer. Stage III pancreatic cancer is an unresectable tumor due to neural invasion without distant metastasis. Based on the severity of neural invasion on computed tomography (CT), patients with stage III pancreatic cancer were divided into high N-inv (n = 14) and low N-inv (n = 4) groups (Supplementary Table 3). Circulating BCAA and BUN levels were significantly higher in the high N-inv group than in the low N-inv group (Supplementary Table 4). In addition, skeletal muscle index was significantly lower in the high N-inv group than in the low N-inv group after 3 months. Overall, elevated plasma BCAAs and the onset of protein degradation are common features of patients with stage III pancreatic cancer with high N-inv and in fasting mice in the N-inv *type severe* group.

### Prognostic effect of hepatic BCKDH in pancreatic cancer patients

**Fig. 4:**
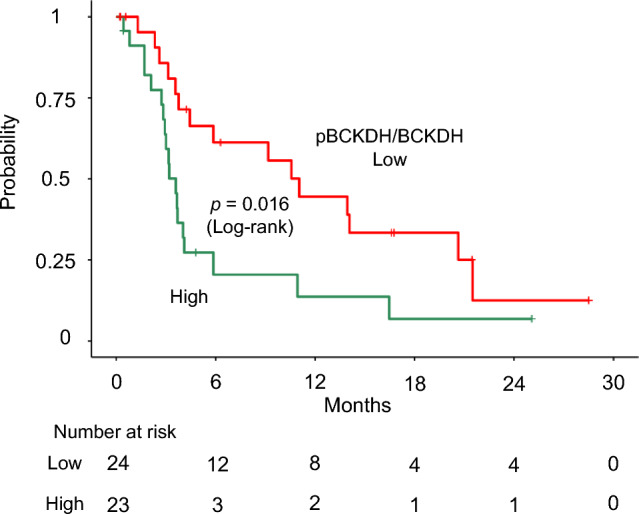
Overall survival according to the status of hepatic BCKDH phosphorylation in patients with stage IV pancreatic cancer. A Kaplan–Meier curve of overall survival and hazard ratio for patients with pancreatic cancer is shown. Ticks on the curves indicate censored observations. The *p*-values of the log-rank test are shown between the curves. The number of at-risk individuals at each time point is also indicated. BCKDH, branched-chain α-keto acid dehydrogenase; pBCKDH, phosphorylated BCKDH; HR, hazard ratio.

In this study, we investigated the clinical relevance of hepatic BCKDH phosphorylation. Hepatic BCKDH phosphorylation was measured using noncancerous liver biopsy samples from 47 patients with pancreatic cancer and liver metastases (Supplementary Table 5). Patients were stratified into two groups according to the level of hepatic BCKDH phosphorylation, using the median value as the threshold. Notably, the overall survival time was poorer in the group with high hepatic BCKDH phosphorylation (N = 24) than in the group with low hepatic BCKDH phosphorylation (N = 23; log-rank *p* = 0.016) (Fig. 4).

## Discussion

Increased plasma BCAA levels due to suppressed activity of hepatic BCKDH have been reported in several diseases, including obesity and diabetes^11,12^. In this study, we showed possible hepatic BCKDH suppression by phosphorylation also exists in pancreatic cancer using the N-inv *type severe* model (Fig. 2A,B). Overall, these findings suggest that the elevated plasma BCAA levels in N-inv *type severe* due to suppressed BCKDH activity in the liver. In addition, the accumulation of BCKAs in the liver (Fig. 2C,D) and reduced whole-body leucine flux in the N-inv *type severe* group (Fig. 2E,F) support the presence of hepatic BCKDH dysfunction and defective BCAA utilization in N-inv *type severe* group. Moreover, the expression levels of *Bcat2*, which encodes an enzyme catalyzing the first step of BCAA catabolism, was significantly lower in the white adipose tissue of mice in the N-inv *type severe* group than in those in the N-inv *type moderate* group (Supplementary Fig. 4B). Collectively, the downregulation in *Bcat2* expression in adipose tissue and the decreased white adipose tissue weight (Supplementary Fig. 2 C) may partially contribute to the elevated plasma BCAA levels in the N-inv *type severe* group. However, the contribution of white adipose tissue to whole-body BCAA oxidation is unclear^3,12^. Given that the rate-limiting BCAA catabolic enzyme is BCKDH^4^ and its oxidative capacity is the highest in the liver in rodents^19^, we believe hepatic BCKDH impairment contributes more to the increased plasma BCAA levels than does suppressed *Bcat2* expression in white adipose tissue in the N-inv *type severe* group. BCAAs are also involved in protein synthesis in animals^2^. Given that protein synthesis activity is relatively low in fasted state—elevated BCAA levels were observed in this condition—we assessed whether the suppressed protein synthesis in N-inv *type severe* group may be responsible for the increased BCAA level by measuring the phosphorylation of hepatic ribosomal S6 protein—a marker of protein synthesis^20^ (Supplementary Fig. 5 A, B). Importantly, the N-inv *type severe* show increased phosphorylation of ribosomal S6 protein, which may be due to BCAA upregulation-mediated activation of mTOR signaling and enhanced ribosomal S6 protein phosphorylation^20^. Overall, these results suggest that protein synthesis is not suppressed in N-inv *type severe* group and that the suppression of BCAA oxidation contributes to increased plasma BCAA levels.

In addition, the N-inv *type severe* group had high plasma levels of BCAA and BUN and upregulated expression of *Murf1*, which are markers of muscle loss in the gastrocnemius (Fig. 3 and Supplementary Fig. 2 C), as well as lower muscle bundle area in the gastrocnemius than the other groups (Fig. 3). Collectively, these findings confirmed that mice in the N-inv *type severe* group were in the initiation phase of muscle loss. Approximately 50% of patients with pancreatic cancer experience cachexia throughout their clinical course^21^. Cancer cachexia causes sarcopenia due to a hypercatabolic state driven by reduced food intake and abnormal metabolism^22^. Plasma BCAA levels increased during the onset of pancreatic cancer^15^ and then decrease during cancer cachexia^23^. Therefore, plasma BCAA levels are speculated to decrease with disease progression. According to the international consensus on cachexia stages, cancer cachexia is classified as pre-cachexia, cachexia, or refractory cachexia^22^. Collectively, the features observed in N-inv *type severe* in this study may be typical of the pre-cachexia stage, which is characterized by elevated plasma BCAA levels and the onset of muscle loss. Moreover, the metabolic feature of N-inv *type severe* group (elevated plasma BCAA and BUN concentrations together with the future onset of skeletal muscle loss) was observed in patients with stage III pancreatic cancer with high N-inv (Supplementary Table 4), suggesting physiological relevance.

Increased hepatic BCKDH phosphorylation in pancreatic cancer was strongly associated with poor prognosis in pancreatic cancer, indicating the clinical significance of reduced BCAA catabolism (Fig. 4). Further studies are required to elucidate the mechanisms underlying the association between upregulated hepatic BCKDH phosphorylation and poor prognosis. One of the potential mechanisms might involve BCKA accumulation due to BCKDH dysfunction in the liver, as observed in the N-inv *type severe* group. Elevated BCKA levels due to impaired BCAA oxidation in the liver has been reported to trigger macrophage hyper-activation and promote chronic inflammation and tissue damage due to mitochondrial oxidative stress in *db/db* mice^24^. Therefore, BCKA accumulation due to impaired hepatic BCKDH in pancreatic cancer with neural invasion could potentially induce chronic inflammation and oxidative stress in metabolic organs that are often associated with poor prognosis in patients with advanced cancer^25–27^.

Despite these promising findings, this study has some limitations. For example, we used only the N-inv model and did not validate our results using other models. Previous studies have used KPC mice as a model of muscle loss in pancreatic cancer^15^. However, we did not use these mice because we considered them unsuitable for studying metabolism in pancreatic cancer. Altered exocrine pancreatic enzymatic function has been identified as a cause of tissue wasting in KPC mice^28^. A prospective double-blind, randomized, placebo-controlled phase II trial was conducted to assess whether pancreatic exocrine replacement therapy can reduce or prevent weight loss in patients with unresectable pancreatic cancer. However, no benefit of pancreatic exocrine replacement therapy has been observed in terms of weight loss suppression^29^. Based on these clinical results, we used an N-inv murine model rather than the KPC model. To establish additional pre-cachectic pancreatic cancer mouse models, screening for tumor cell clones^30^ that exhibit a pre-cachectic phenotype may be a viable approach. In addition, we used ^15^N-leucine to quantify whole leucine flux. More appropriately, an infusion test using ^13^C-BCKA label should be performed to quantify BCKA oxidation through BCKDH. Furthermore, the molecular mechanisms underlying the upregulation of hepatic BCKDH phosphorylation and its association with poor prognosis in pancreatic cancer remain unclear. Finally, we did not evaluate the association between hepatic BCKDH phosphorylation and circulating BCAAs or BCKAs in patients with pancreatic cancer in this study.

Overall, our study demonstrated that the possible presence of suppressed BCAA catabolism in the liver was due to the downregulation of hepatic BCKDH in the pre-cachexia stage of pancreatic cancer. This defective hepatic BCAA utilization was linked to the onset of muscle loss and poor prognosis in patients, highlighting its strong clinical impact. Further studies are warranted to investigate the clinical features and alterations in hepatic metabolism in patients with pancreatic cancer with elevated hepatic BCKDH phosphorylation.

## Materials and methods

### Ethical approval

This clinical component was an observational study and was approved by the National Cancer Center Institutional Review Board (approval no. 2017–200) and the Institutional Review Board of Ajinomoto, Inc. (approval no. 2017–018), and complied with the Declaration of Helsinki and the Japanese Ethical Guidelines for Medical and Health Research Involving Human Subjects. Animal studies were conducted at Ajinomoto Inc. in accordance with the approved Institutional Animal Care and Use Committee (IACUC) protocol. Although this animal study was not conducted in accordance with the Food and FDA Good Laboratory Practice regulations, 21 CFR Part 58, all experimental data management and reporting procedures were performed in strict accordance with Ajinomoto, Inc. Guidelines and Standard Operating Procedures. All animal experimental procedures were approved by the Animal Study Ethics Committee of Ajinomoto Co., Inc. and followed the institutional guidelines of Ajinomoto Co., Inc. (approval nos. 2020145 and 2,021,061). The animal studies were designed and reported in accordance with the ARRIVE guideline.

### Clinical study

Participants were prospectively registered between August 2011 and January 2015 at the National Cancer Center Hospital East after providing written informed consent. Eligible patients in this study had treatment-naïve pancreatic cancer with stage III tumors, according to the TNM classification of the International Union Against Cancer, or liver metastasis. All tumors were pathologically diagnosed as pancreatic cancer. Plasma and clinical data were collected before and three months after anti-cancer treatment. Lumbar skeletal muscle mass at the level of the third lumbar vertebra (L3) was measured by analyzing electronically stored CT images using Slice-O-Matic software (version 5.0; Tomovision, Montreal, Quebec, Canada). The sum of the cross-sectional areas (cm^2^) of the muscles in the L3 region was normalized to height (m^2^) and reported as the lumbar skeletal muscle index (cm^2^/m^2^). In addition, a tissue core was obtained from non-cancerous liver tissue during the collection of biopsy samples from liver metastases for pathological diagnosis before anti-cancer treatment. Liver lysates were obtained after protein purification using a NucleoSpin TriPrep kit (Macherey–Nagel, GmbH & Co.KG, Düren, Germany). Thereafter, proteins were separated using an SDS-PAGE sample prep kit (Thermo Fisher Scientific, Massachusetts, USA) and stored at − 80 °C until use, as previously described^31^. All purified protein lysates were tested in duplicate to evaluate β-actin expression using a mouse monoclonal antibody (8H10D10, Cell Signaling Technology, Danvers, MA, USA) with the Jess™ Simple Western automated nano-immunoassay system (ProteinSimple, San Jose, CA, USA, a Bio-Techne Brand)^32^. Patients from whom liver lysates showed unstable β-actin expression were excluded from this study. Protein lysates with stable β-actin expression were evaluated using primary antibodies against total BCKDH (A303-789A, Bethyl Laboratories, Inc., Texas, USA) and phosphorylated BCKDH (A304-672A, Bethyl Laboratories, Inc.). The phosphorylated BCKDH/BCKDH ratio was calculated for each patient.

In patients with stage III tumors, the degree of N-inv was classified as high or low based on the severity of perivascular soft tissue (PVST) around the superior mesenteric artery (SMA) and celiac artery (CeA) on CT images. Low N-inv was defined as PVST intact to neither the SMA nor the CeA. PVST in contact with the SMA or CeA was defined as high N-inv.

### Cell culture

Capan-1 cells, a human pancreatic cancer cell line, were purchased from the American Type Culture Collection (HTB-79™). Briefly, the cells were cultured in Iscove’s modified Dulbecco’s medium (1,244,053, Thermo Fisher Scientific) supplemented with 20% heat-inactivated fetal bovine serum (172,012, Nichirei Biosciences Inc., Tokyo, Japan) and 1% penicillin/streptomycin (15,140–122, Thermo Fisher Scientific) and incubated at 37 °C under a 5% CO_2_ atmosphere.

### Animal model

Eight-week-old male severe combined immunodeficiency (SCID) mice (Clea Japan, Tokyo, Japan) were housed in light- and temperature-controlled rooms (temperature, 23 ± 1 °C; 12 h/12 h dark cycle) and provided with standard food ad libitum (CRF-1, Oriental Yeast, Tokyo, Japan). Neural invasion (N-inv) and subcutaneous tumor (SC) models were generated using Capan-1 cells, as previously described^16^. For the N-inv model, Capan-1 cells (2.5 × 10^4^) were injected into the left sciatic nerve. For the SC model, cells were injected into the subcutaneous tissue of the left flank. Mice in the sham control group were injected with phosphate-buffered saline (PBS). During the study period, body weight was monitored weekly. All mice were fasted overnight and sacrificed at 16 weeks of age. Body and tumor weights of the mice at the time of sacrifice are shown in Supplementary Table 1 and Supplementary Table 2 for animals in cohorts 1 and 2, respectively (see below). All mice were euthanized by exsanguination via transection of the inferior vena cava under deep anesthesia with 3.5% isoflurane. Details of plasma sample collection are provided in a subsequent section. Tumors, livers, kidneys, right gastrocnemius muscles, epididymal white adipose tissue, and 13^th^ thoracic spinal cord were harvested, weighed, and snap-frozen in liquid nitrogen or soaked in RNAlater™ Stabilization Solution (AM7020, Invitrogen, Massachusetts, USA) for RNA isolation and then snap-frozen. Non-frozen tissues comprising the tumor and spine were fixed in 4% paraformaldehyde phosphate buffer solution (09,154–85, Nacalai Tesque, Kyoto, Japan), embedded in paraffin, and cut into thin sections. Thereafter, N-inv tumor sections were stained with hematoxylin and eosin to evaluate perineural invasion, during which cancer cells existed in the perineural space, formed a ductal structure, and adhered to the nerve bundle or perineurium^33^. Spinal sections were stained with an antibody against mouse GFAP (1/2000, Millipore, Billerica, MA, USA) to evaluate spinal neuroinflammation in the dorsal horn^16^. Notably, the N-inv model was considered successfully induced if both perineural invasion and spinal neuroinflammation were detected.

### Animal cohorts

We generated two animal cohorts, referred to as animal cohorts 1 and 2. Notably, the primary difference between the two cohorts was whether an infusion test (see below) was performed and whether sham control models were generated. The infusion test was conducted only in animal cohort 2. Based on animal welfare concerns and to reduce the number of mice used in the experiments, sham control groups were not created in animal cohort 2. Parameters evaluated in each cohort are listed in Supplementary Table 6. Blood samples from mice in cohort 1 were collected from the vena cava using a 100 mg/mL EDTA/2Na-coated syringe. In cohort 2, blood samples for plasma NEFA and BUN measurements were collected from the vena cava at the end of the infusion test, using syringes coated with 100 mg/mL EDTA-2Na, whereas blood samples for the other analyses were collected from the tail vein immediately before the infusion test, using capillary tubes coated with 100 mg/mL EDTA-2Na. After collection, the blood samples were immediately placed on ice and centrifuged (1,600 × *g*, 4 °C, 15 min) to collect the plasma, which was stored at − 80 °C.

### Classification of N-inv *type severe* and N-inv* type moderate*

Mice in the N-inv group were classified as N-inv *type severe* and *moderate* based on plasma BCAA levels using k-means classification. This classification was performed using the kmeans() function in R version 4.0.5 with the option “centers = 2.”

### Measurement of cross-sectional areas of the muscle fibers

Briefly, gastrocnemius muscle sections were stained with hematoxylin and eosin to evaluate the cross-sectional areas of skeletal muscle fibers using light microscopy. The number of muscle fibers was counted along two randomly drawn lines perpendicular to the long axis of the muscle fibers, and the average diameter of a single muscle fiber was calculated. Thereafter, the cross-sectional areas of the muscle fibers were determined using the following formula: cross-sectional area of the muscle fibers = (average diameter of muscle fibers × 1/2) ^2^ × 3.14.

### Amino acid quantification

Briefly, tissue samples (10 mg) were homogenized in 1 mL of 80% methanol (21914–45, Nacalai Tesque, Kyoto, Japan)/water with 6 μM Phe-d5. Thereafter, 400 µL of the homogenized sample was mixed with 400 μL of chloroform (036–01926, FUJIFILM Wako Pure Chemical Corporation, Osaka, Japan) and 400 μL of water and centrifuged to collect the aqueous phase. Plasma samples were mixed with an internal standard solution and acetonitrile and then centrifuged to collect the supernatants. Both tissue and plasma samples were prepared for LC–MS/MS analysis using an APDSTAG® Wako Amino Acids Internal Standard Mixture Solution (293–73701, FUJIFILM Wako Pure Chemical Corporation, Osaka, Japan). Analysis was performed on an Agilent 6495 Triple Quad LC/MS system using specific columns, and the data were processed using Mass Hunter software and Microsoft Excel 365. Further details are provided in the Supporting Information.

### Infusion test using stable isotope-labeled ^15^N-leucine

Briefly, catheters were surgically implanted into the right jugular veins of mice under anesthesia 7 weeks after Capan-1 cell injection, as described previously^34^. After recovering for at least 3–5 days, the mice were fasted for 24 h and infused with a BCAA solution via a catheter based on 15 min of priming (2.76 mmol/kg/h, 18 μL/min) followed by 4 h at a constant rate (0.46 mmol/kg/h, 2 μL/min) in a conscious state. The BCAA solution was prepared by dissolving ^15^N-leucine (NLM-142-PK, Cambridge Isotope Laboratories, Inc. Massachusetts, USA), isoleucine (Ajinomoto Co., Inc., Kawasaki, Japan), and valine (Ajinomoto Co., Inc.) in saline (Otsuka Pharmaceutical Factory, Inc., Tokyo, Japan) at a 2:1:1 concentration ratio. Blood samples were collected from the tail vein using EDTA-coated capillary tubes immediately before and every hour after infusion.

### Data analysis of amino acid labeling ratio

The rate of appearance (Ra) of total endogenous (unlabeled) leucine was calculated based on the plasma sample data using the following steps:

1. The *tracer*-*to*-*tracee ratio* (TTR) was calculated as follows:
  1 $$TTR = \frac{{\left( {area \:of \:labeled \:leucine} \right)}}{{\left( {area \:of \:unlabeled \:leucine} \right)}} - \frac{{\left( {area \:of \:labeled \:leucine \:of \:time \:0} \right)}}{{\left( {area \:of \:unlabeled \:leucine \:of \:time \:0} \right)}}$$
2. The *mole percent excess* (MPE) was calculated as follows:
  2 $$MPE = \frac{TTR}{{1 + TTR}}$$
3. MPE was calculated at each time point (0, 1, 2, 3, and 4 h after the initiation of infusion).
4. The mathematical model shown as below was fitted to the blood collection time t and MPE y for each mouse.
  3 $$y\left( t \right) = Y_{0} + \left( {Plateau - Y_{0} } \right)(1 - e^{ - kt} )$$
5. Ra was calculated by dividing the infusion rate by the optimized parameter *Plateau*.

Parameter optimization was performed using the R package “optimx.” If the MPE of a mouse at 4 h after infusion initiation was < 0.9 × the optimized parameter *Plateau* or the R^2^ of the fitted model was < 0.5, the data of the infusion test of the mouse were excluded from the analysis because the former condition indicates that the MPE did not reach an equilibrium state, and the latter indicates poor fitting of the model. Based on this criterion, 1 of the 22 mice used for the infusion test was eliminated from the analysis.

### Quantification of liver BCKA concentrations

Briefly, murine liver samples were added to a 2.0 mL tube containing zirconia beads (Bio Medical Science Inc., Tokyo, Japan) and 800 μL of 100% acetonitrile, with 10 μL of 0.5 mg/mL of 2-isopropyl malic acid and a volume of water adjusted based on the sample weight. Thereafter, the samples were homogenized and incubated before centrifugation to collect the supernatants for analysis. To quantify BCKAs, samples were prepared over a concentration range and subjected to solid-phase derivatization. Thereafter, the derivatized analytes were analyzed using a GCMS-TQ8050 system equipped with a BPX-5 capillary column. The GC–MS parameters were set to optimize the separation and detection of metabolites, with identification supported by the Smart Metabolites Database. For low-concentration samples, additional dilutions were prepared and quantified. Further details on sample preparation and GC–MS analysis are provided in the Supporting Information.

### Blood chemistry tests

Plasma NEFA and BUN levels were measured using the NEFA C-Test (FUJIFILM Wako Pure Chemical Corporation) and Fuji DRY-CHEM (Fujifilm, Tokyo, Japan), respectively. Blood samples used for the infusion test were collected immediately before the initiation of the test. Plasma glucose and ketone levels were measured using FreeStyle Precision Neo Blood Glucose and Ketone Monitoring System (Abbott, Illinois, USA).

### qRT-PCR

Total RNA was extracted using the RNeasy Plus Universal Mini Kit (73,404, Qiagen, Hilden, Germany) and reverse-transcribed to generate cDNA using PrimeScript™ RT Master Mix (RR036A, Takara Bio, Kusatsu, Japan). qRT-PCR was performed on a Quant Studio™ 12 K Flex (Applied Biosystems) using Power SYBR™ Green PCR Master Mix (4367659, Applied Biosystems, Massachusetts, USA). The PCR conditions were as follows: initial pre-incubation at 95 °C for 10 min, followed by 40 cycles of incubation at 95 °C for 15 s and incubation at 60 °C for 1 min. Gene expression was normalized using the ΔΔCT method with *cyclophilin* as the reference. All primers are listed in Supplementary Table 7.

### Western blot analysis

Murine snap-frozen liver samples were lysed with RIPA buffer (08714, Nacalai Tesque) containing a phosphatase inhibitor cocktail (07574, Nacalai Tesque) and a protease inhibitor cocktail (25955, Nacalai Tesque), using a multi-bead shocker (Yasui Kikai, Osaka, Japan) at 2000 rpm for 10 s. Thereafter, the protein concentration was quantified using a Pierce BCA Protein Assay Kit (23225, 23227, Thermo Fisher Scientific). The lysed liver samples were mixed with Laemmli Sample Buffer (1610737, Bio-Rad, California, USA) containing 5% 2-mercaptoethanol and boiled at 95 °C for 5 min. Thereafter, 5 μg of protein per well was loaded onto a Criterion TGX gel 10% (5671034J10, Bio-Rad), electrophoresed, and transferred to PVDF membranes (162–0177, Bio-Rad). After blocking with Starting Block™ (PBS) Blocking Buffer (37538, Thermo Fisher Scientific) for 30 min, the membranes were incubated with the respective primary antibodies (diluted 1:1000), including anti-total BCKDH (A303-789A, Bethyl Laboratories, Inc.), anti-phosphorylated BCKDH (A304-672A, Bethyl Laboratories, Inc.), anti-total ribosomal S6 protein (2217, Cell Signaling Technology), anti-phosphorylated ribosomal S6 protein (4858, Cell Signaling Technology), and anti-α tubulin (2144, Cell Signaling Technology) overnight at 4 °C. Thereafter, the membranes were incubated with a secondary antibody (7074, Cell Signaling Technology, 1:5000 dilution) for 1 h at room temperature (23 °C). Signals were detected using Chemi-Lumi One L (07,880, Nacalai Tesque) for total BCKDH or Chemi-Lumi One Ultra (11,644, Nacalai Tesque) for the others and images were captured using an Amersham Imager 600 (GE HealthCare, Chicago, USA). The results were quantified using the built-in software of the Amersham Imager 600. α-Tubulin data were obtained by reblotting using Western BLoT Stripping Buffer (T7135A, Takara Bio).

### Statistical analyses

Significant differences between groups were determined using one-way ANOVA with Dunnett’s post hoc test (multi-group comparisons) or two-tailed unpaired Student’s t-test (two-group comparisons) in R package. Patients with stage IV pancreatic cancer were divided into high- and low-pBCKDH/BCKDH groups, based on the dichotomized pBCKDH/BCKDH status (≥ or < median). Overall survival was calculated from the start date of first-line chemotherapy or best supportive care. Survival curves were estimated using the Kaplan–Meier method. Differences between the survival curves were compared using the log-rank test. Survival analysis was performed using JMP version 11 (SAS Institute, Cary, NC, USA), whereas all other analyses were performed using R 4.4.3. Statistical significance was set at *p* < 0.05. Data are presented as mean ± standard deviation.

## Supplementary Information

Supplementary Information.

## Data Availability

Qualified researchers can contact Shuichi Mitsunaga to request individual-level patient data. The datasets from the animal experiments in the current study are available from Asami Hagiwara upon reasonable request.
