# Supplementary material for: Branched-chain amino acid catabolism is potentially deficient in pancreatic cancer with high-neural invasion
Source: Sci Rep. 2026 Apr 12;16:17012. doi: 10.1038/s41598-026-47741-x (PMC13230552; doi:10.1038/s41598-026-47741-x)
Supplement: Supplementary file 1 — Supplementary Information. [file 41598_2026_47741_MOESM1_ESM.pdf]

Supplementary Materials for

Branched-chain amino acid catabolism is potentially deficient in pancreatic cancer with high-neural invasion

Hiroki Michida[a,1], Asami Hagiwara[a,1], Yuki Saito[a], Noriko Kawasaki[a], Mayuka Kanda[a], Mika Kawasaki[a], Sachise Karakawa[a], Hidetaka Suzuki[b,c], Masafumi Ikeda[d], Hideki Makinoshima[e,f], Shuichi Mitsunaga[b,d,*]

[a] Research Institute for Bioscience Products and Fine Chemicals, Ajinomoto Co., Inc., Kawasaki, Japan

[b] Division of Biomarker Discovery, Exploratory Oncology Research & Clinical Trial Center, National Cancer Center, Kashiwa, Japan

[c] Department of Pharmacy, National Cancer Center Hospital East, Kashiwa, Japan

[d] Department of Hepatobiliary and Pancreatic Oncology, National Cancer Center Hospital East, Kashiwa, Japan

[e] Tsuruoka Metabolomics Laboratory, National Cancer Center, Tsuruoka, Japan

[f] Division of Translational Informatics, Exploratory Oncology Research and Clinical Trial Center, National Cancer Center, Kashiwa, Japan

[1]These authors contributed equally to this work.

* Shuichi Mitsunaga, MD, PhD, Division of Biomarker Discovery, Exploratory Oncology Research & Clinical Trial Center, National Cancer Center, Kashiwanoha 6-5-1, Kashiwa, Chiba 277-8577, Japan.

Email: smitsuna@east.ncc.go.jp; **Tel:** +81-4-7133-1111; **Fax:** +81-4-7133-6865

Detailed materials and methods

Amino acid quantification

Crushed tissue samples (10 mg) were suspended in 1 mL of homogenate solution (80% [v/v] methanol [21914-45, Nacalai Tesque]/water, 60 µM Phe-d5) and homogenized using a Precellys homogenizer (Bertin Technologies, Montigny-le-Bretonneux, France) at 6000 rpm for 10 s twice, with a 5-s interval. Thereafter, a mixture comprising 400 µL of the homogenized sample, 400 µL of chloroform (036-01926, FUJIFILM Wako Pure Chemical Corporation, Osaka, Japan), and 400 µL of pure water was centrifuged at 20,000 ×*g* for 5 min at 4 °C to collect the aqueous phase for amino acid quantification.

Plasma samples (10 µL) were mixed with 10 µL of the internal standard solution and 20 µL of acetonitrile. Thereafter, the mixture was centrifuged at 20,000 ×*g* for 10 min at 20 °C to collect the supernatant.

For LC-MS/MS sample preparation, the APDSTAG® Wako Amino Acids Internal Standard Mixture Solution (293-73701, FUJIFILM Wako Pure Chemical Corporation) was added to the extracted tissue amino acid solution or plasma samples. LC-MS/MS analysis was performed using an Agilent 6495 Triple Quad LC/MS G6495B (Agilent Technologies, Inc., California, USA) with an Inertsil ODS-3 1.5 × 10 mm, 3 µm (GL Science, Tokyo, Japan) as the guard column and an Inertsil C8-3 2.1 × 100 mm, 3 µm (GL Sciences) or Shim-pack UF-AMINO 2.1 × 100 mm, 2 µm (Shimazu Corporation, Kyoto, Japan) as the analysis column. Mass Hunter workstation software (Agilent Technologies, Inc.) and Microsoft Excel 365 were used for data processing.

Quantification of BCKA concentrations in the liver

Mouse liver samples were added to a 2.0 mL tube containing zirconia beads (Bio Medical Science Inc., Tokyo, Japan), 800 µL of 100% acetonitrile (ACN), 10 µL of 0.5 mg/mL of (Milli-Q) 2-isopropyl malic acid, and X µL of water (where X = 190 − sample weight [mg]), crushed using a ShakeMaster® AUTO (Bio Medical Science Inc.) at 1,000 rpm for 10 min, and incubated at 37 °C for 30 min. Two zirconia beads (Bio Medical Science Inc.) with a diameter of 5.0 mm and four beads with a diameter of 3.0 mm were added to the mixture in the 2.0 mL tube (Master Tube Hard; Bio Medical Science Inc.). Thereafter, the mixture was centrifuged at 3000 ×*g* for 5 min at 4 °C to collect the supernatant, which was transferred to a 1.5 mL tube and centrifuged at 14,000 rpm for 5 min at 4 °C, and the supernatant was collected into another tube. To quantify the contents of 2-keto-isovaleric acid, 3-methyl-2-oxovaleric acid, and 2-ketoisocaproic acid BCKAs, we prepared samples comprising each compound (0, 50, 100, 500, and 1 µM). The solid phase was derivatized as follows: the Presh-SPE AOS solid-phase cartridge was supplied by AiSTI SCIENCE (Wakayama, Japan). The derivatization conditions were 3 min of methoximation with 5 µL of 20% methoxyamine solution and 1 min of trimethylsilylation with 25 µL of N-methyl-N-trimethylsilyl-trifluoroacetamide. Derivatized analytes were effectively eluted with 100 µL of n-hexane, and 1.0 µL of the derivatized solution was injected into the gas chromatograph/mass spectrometer GCMS-TQ8050 (Shimadzu Corporation). A calibration curve was created using the internal standard method with GC-MS solution software (Shimadzu Corporation), and quantification was performed. For the standard preparation, a 10-fold concentrated aqueous solution was prepared (Milli-Q). Thereafter, 100 µL of the aqueous solution, 800 µL of 100% ACN, 10 µL of 0.5 mg/mL (Milli-Q) 2-isopropyl malic acid, and 90 µL of Milli-Q water were added to zirconia beads (Bio Medical Science Inc.), which were transferred to a 2-mL tube; the same treatment was performed as that for the sample measurement. For samples with a detected concentration of 50 nM or less, new preparations were performed using 0, 5, 10, and 50 nM of each compound.

Metabolomic analysis was performed using a GCMS-TQ8050 equipped with a BPX-5 capillary column (internal diameter: 30 m × 0.25 mm; film thickness: 0.25 μm; SEG, Victoria). The parameter settings were as previously described [1]. During GCMS-TQ8050 analysis, the inlet temperature was maintained at 250 °C, and helium was used as the carrier gas at a constant flow rate of 39 cm/s. The injector split ratio was 1:10. The GC column temperature was programmed as follows: 60 °C for 2 min, an increase from 60 to 330 °C at a rate of 15 °C per min, and 330 °C for 3 min. The total GC runtime was 23 min. The transfer-line and ion-source temperatures were 280 and 200 °C, respectively. The ionization voltage was set to 70 eV. To increase the detection sensitivity, the detector voltage for sensing ions was changed from 0.5 to 0.8 kV. Argon was used as the collision-induced dissociation gas. Metabolites were detected using the Smart Metabolites Database (Shimadzu Corporation), which contains the relevant MRM method file and data regarding the GC analytical conditions, MRM parameters, and the retention index employed for metabolite measurements. The Automatic Adjustment of Retention Time (AART) function of GC-MS solution software (Shimadzu Corporation) and a standard alkane series mixture (C7 to C33) were used to correct the retention time. Peaks were identified automatically and confirmed manually based on the specific precursor, product ions, and retention time.

References

[1] Nishiumi, S. et al. Investigations in the possibility of early detection of colorectal cancer by gas chromatography/triple-quadrupole mass spectrometry. *Oncotarget* **8**, 17115-17126 (2017).

Supplementary Figures and Tables

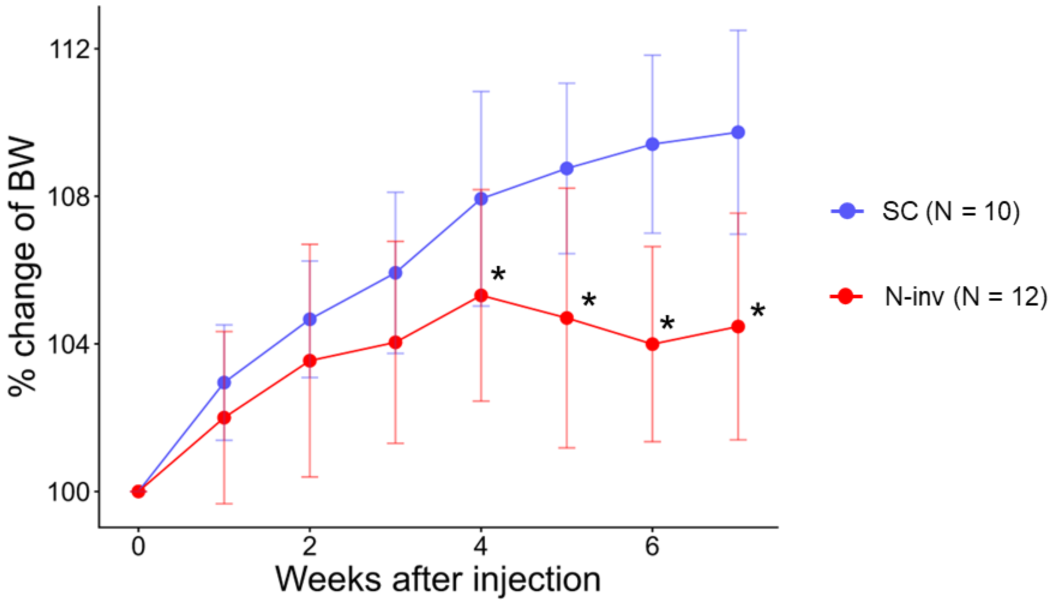

Supplementary Fig. 1. Percentage change in body weight of mice in cohort 2. Representative data are shown as the mean ± standard deviation. SC, subcutaneous tumor model; N-inv, neural invasion model. *; $p < 0.05$, based on Student's t-test.

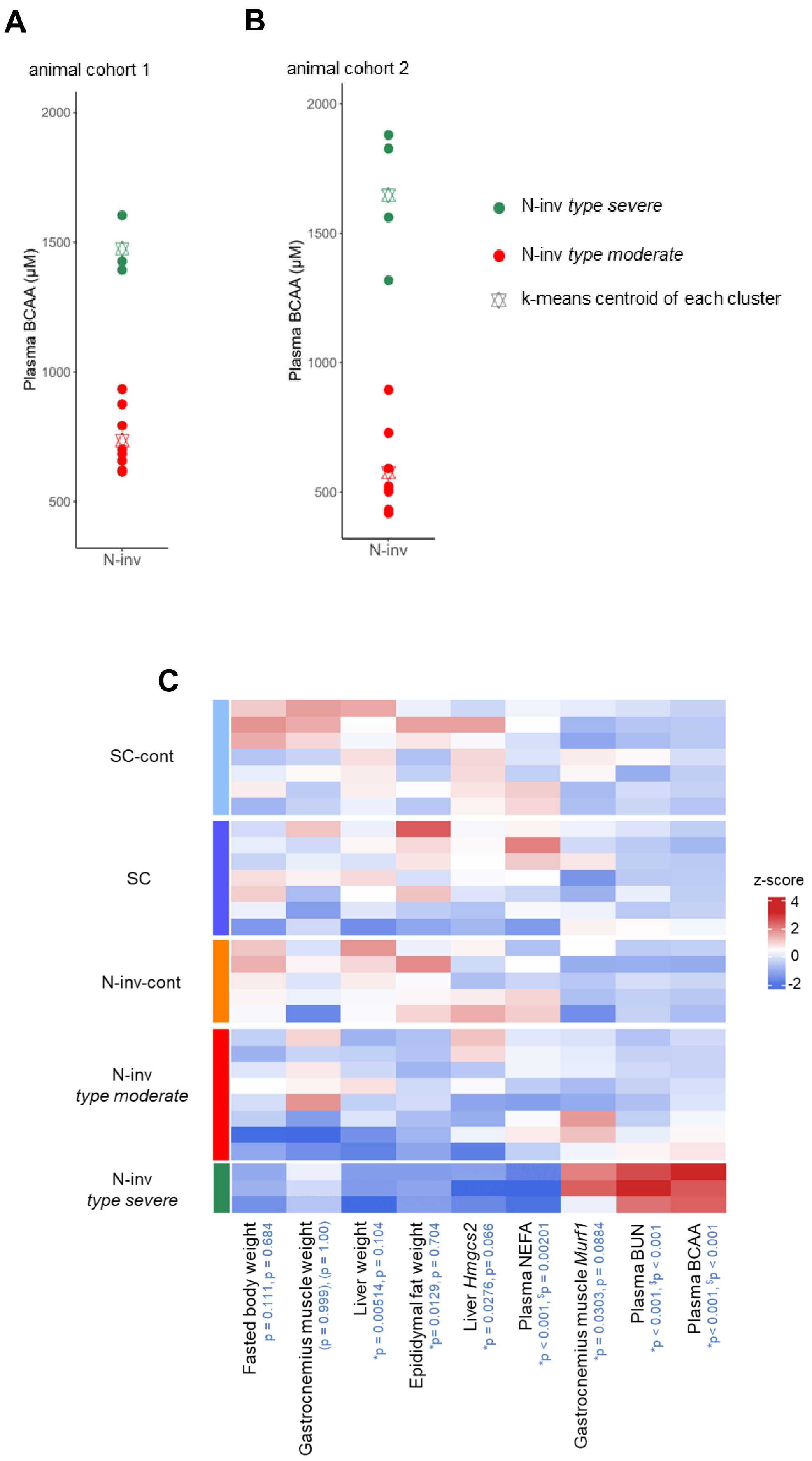

A
B
animal cohort 1
animal cohort 2
Plasma BCAA (μM)
2000
1500
1000
500
N-inv
N-inv type severe
N-inv type moderate
k-means centroid of each cluster
C
SC-cont
SC
N-inv-cont
N-inv
type moderate
N-inv
type severe
z-score
4
2
0
-2
Fasted body weight
p = 0.111, p = 0.684
Gastrocnemius muscle weight
(p = 0.999), (p = 1.00)
Liver weight
*p = 0.00514, p = 0.104
Epididymal fat weight
*p= 0.0129, p = 0.704
Liver Hmgcs2
*p = 0.0276, p= 0.066
Plasma NEFA
*p < 0.001, $p = 0.00201
Gastrocnemius muscle Murf1
*p = 0.0303, p = 0.0884
Plasma BUN
*p < 0.001, $p < 0.001
Plasma BCAA
*p< 0.001, $p < 0.001

Supplementary Fig. 2. Classification of N-inv *type severe* and *moderate* groups and metabolic parameters. (A–B) Classification of N-inv *type severe* and *moderate* groups based on total plasma BCAA levels using the k-means method (A) in mice in cohorts 1 and (B) 2. (C) Heatmap of metabolic parameters of each mouse in cohort 1. Only the right gastrocnemius muscle was weighed because the human pancreatic cancer cell line Capan-1 cells or PBS was injected into the left sciatic nerve or subcutaneous tissue of the left flanks of mice. Epididymal fat was weighed bilaterally. The color indicates the z-score. Two *p*-values based on Dunnett's post-hoc test, for the comparisons of N-inv *type severe* vs. SC and N-inv *type severe* vs. N-inv *type moderate* are shown in blue letters. * and $ indicate statistical significance at $p < 0.05$ for the N-inv *type severe* vs. SC and N-inv *type severe* vs. N-inv *type moderate* comparisons, respectively. The *p*-values calculated based on Dunnett's post hoc test for comparisons that were not significant in the one-way ANOVA are shown in parentheses. SC, subcutaneous tumor model; N-inv, neural invasion model; SC-cont, sham control group for SC; N-inv-cont, sham control group for N-inv; BCAA, branched-chain amino acid; *Hmgcs2*, 3-hydroxy-3-methylglutaryl-CoA synthase 2; NEFA, non-esterified fatty acid; *Murf1*, muscle ring finger protein 1; BUN, blood urea nitrogen.

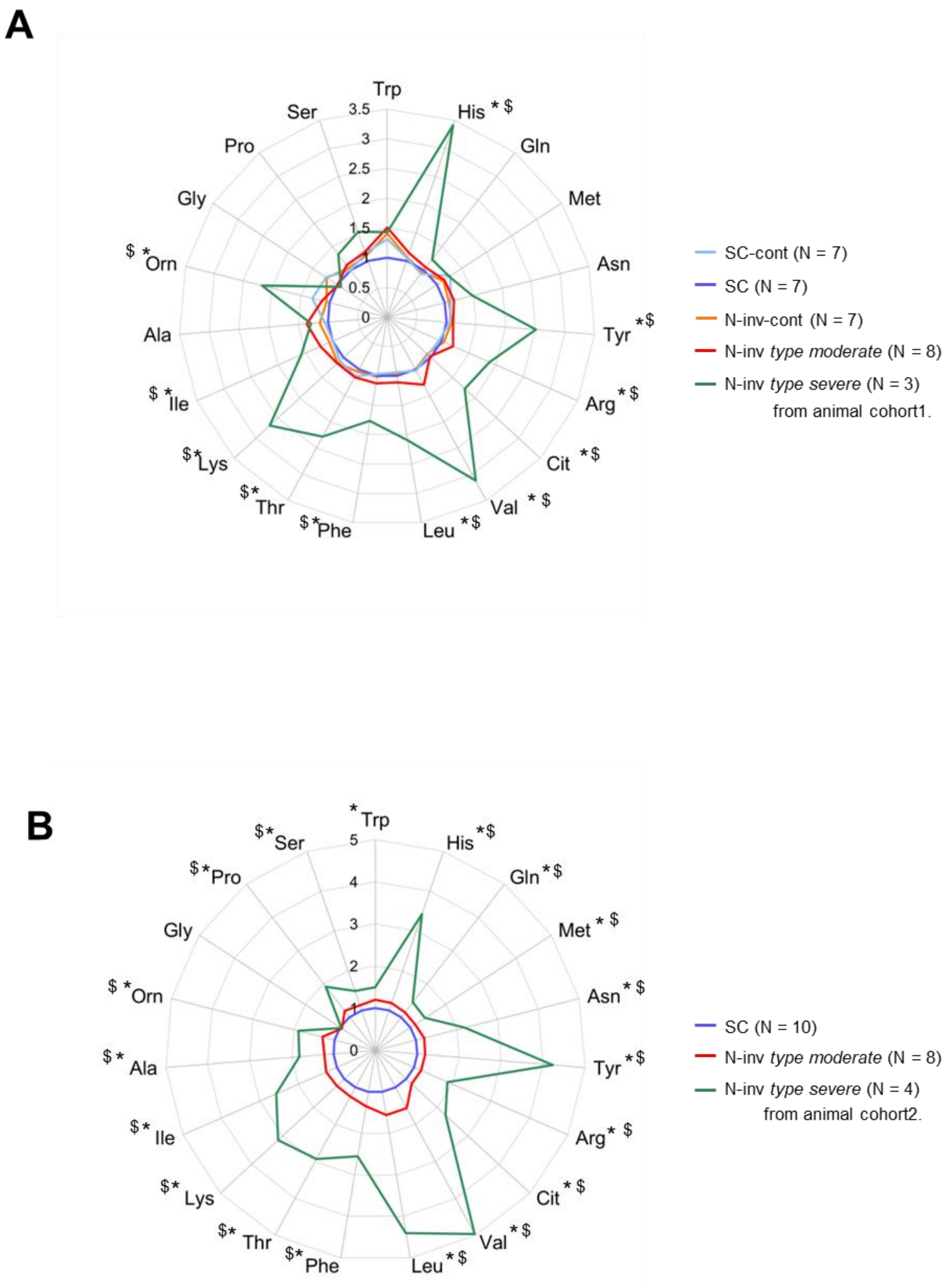
A
Trp
His * $
Gln
Met
Asn
Tyr *$
Arg * $
Cit * $
Val * $
Leu * $
$ * Phe
$* Thr
$* Lys
$ * Ile
Ala
$ * Orn
Gly
Pro
Ser
3.5
3
2.5
2
1.5
1
0.5
0
SC-cont (N = 7)
SC (N = 7)
N-inv-cont (N = 7)
N-inv type moderate (N = 8)
N-inv type severe (N = 3)
from animal cohort1.
B
* Trp
His *$
Gln * $
Met * $
Asn * $
Tyr * $
Arg * $
Cit * $
Val * $
Leu * $
$* Phe
$* Thr
$* Lys
$* Ile
$ * Ala
$ * Orn
Gly
$* Pro
$* Ser
5
4
3
2
1
0
SC (N = 10)
N-inv type moderate (N = 8)
N-inv type severe (N = 4)
from animal cohort2.

Supplementary Fig. 3. Plasma amino acid profile in N-inv *type severe* mouse. Plasma amino acid profile in murine models (A) in cohorts 1 and (B) 2, at 8 weeks after cell injection. Plasma was collected from the vena cava of mice in cohort 1. For cohort 2, plasma for amino acid quantification was collected from the tail vein immediately before the infusion test. Data were normalized to the SC group. "*" and "$" indicate statistical significance at $p < 0.05$ (Dunnett's post-hoc test) for N-inv vs. SC and N-inv vs. N-inv-cont comparisons, respectively.

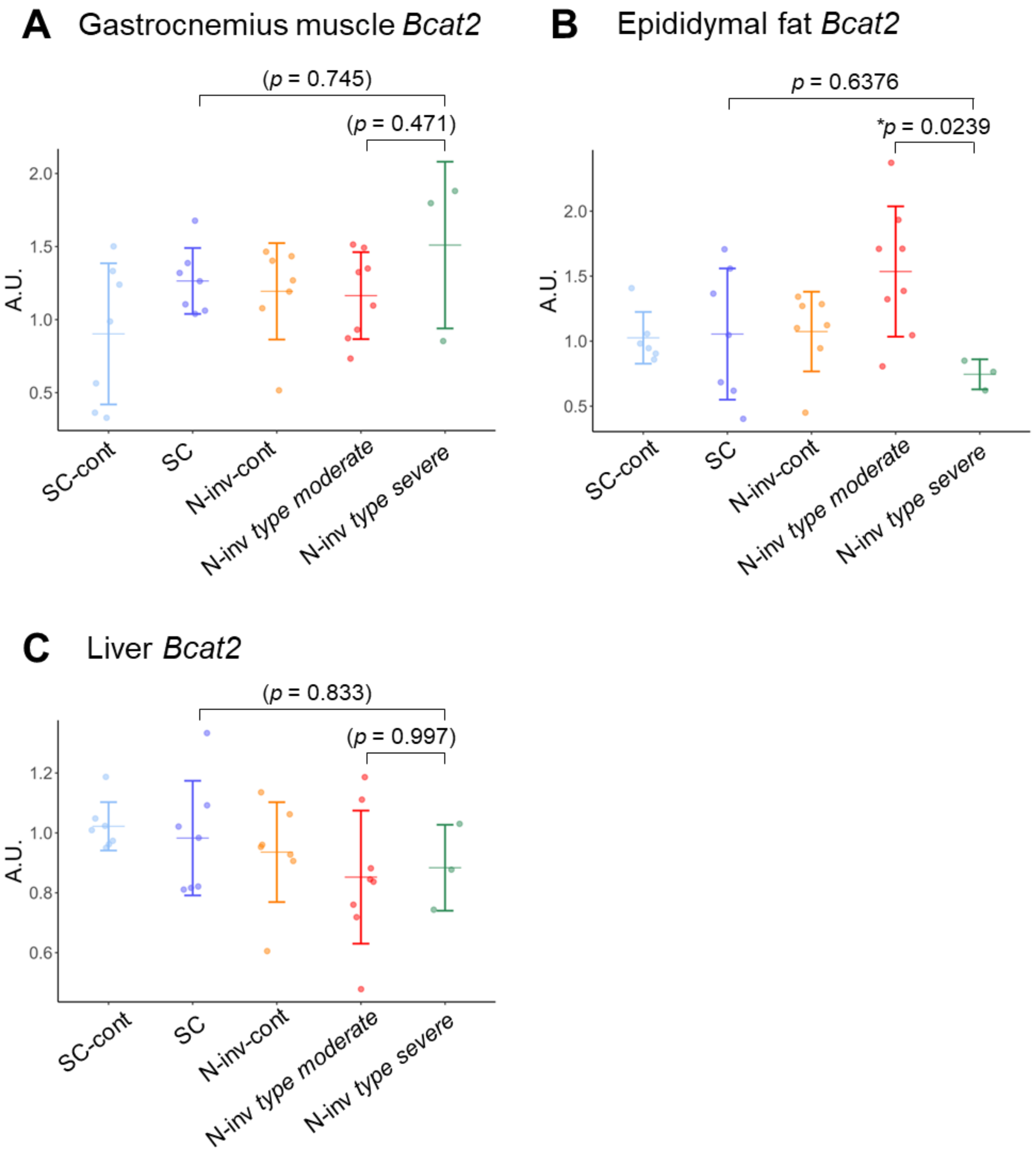
A
Gastrocnemius muscle Bcat2
(p = 0.745)
(p = 0.471)
A.U.
2.0
1.5
1.0
0.5
SC-cont
SC
N-inv-cont
N-inv type moderate
N-inv type severe
B
Epididymal fat Bcat2
p = 0.6376
*p = 0.0239
A.U.
2.0
1.5
1.0
0.5
SC-cont
SC
N-inv-cont
N-inv type moderate
N-inv type severe
C
Liver Bcat2
(p = 0.833)
(p = 0.997)
A.U.
1.2
1.0
0.8
0.6
SC-cont
SC
N-inv-cont
N-inv type moderate
N-inv type severe

Supplementary Fig. 4. *Bcat2* expression levels in various tissues in mouse models. (A–C) *Bcat2* expression levels in the (A) gastrocnemius muscle, (B) epididymal fat, and (C) liver, evaluated in mice in cohort 2. Data are presented as mean ± standard deviation. *$p < 0.05$, based on one-way ANOVA with Dunnett's post-hoc test. The p-values calculated based on Dunnett's post hoc test for comparisons that were not significant in the one-way ANOVA are shown in parentheses. *Bcat2*, mRNA levels of branched-chain amino acid transaminase 2; SC, subcutaneous tumor model; N-inv, neural invasion model; SC-cont, sham control group for SC; N-inv-cont, sham control group for N-inv.

A

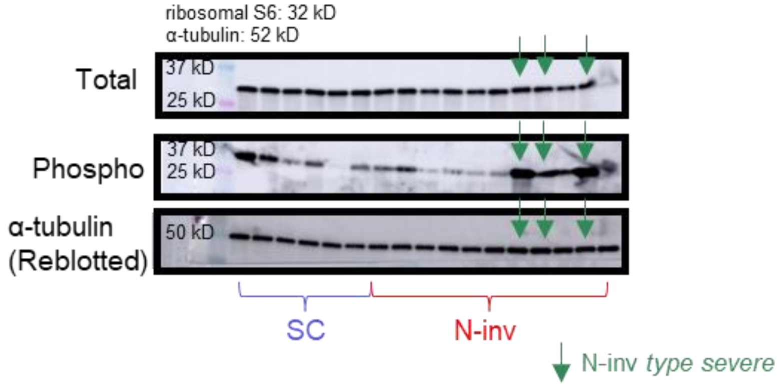

ribosomal S6: 32 kD
α-tubulin: 52 kD
Total
Phospho
α-tubulin
(Reblotted)
37 kD
25 kD
50 kD
SC
N-inv
N-inv type severe

B

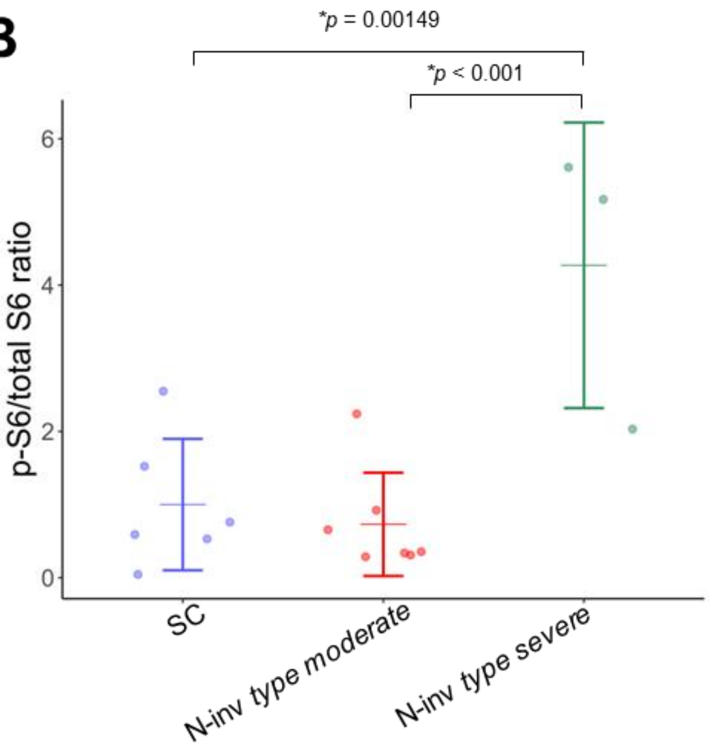

*p = 0.00149
*p < 0.001
p-S6/total S6 ratio
0
2
4
6
SC
N-inv type moderate
N-inv type severe

Supplementary Fig. 5. Phosphorylation level of ribosomal S6 in the liver of mouse models. (A) Western blot images of total liver total ribosomal S6, phosphorylated ribosomal S6, and α-tubulin. The image of α-tubulin is a re-blot. Arrows indicate the bands for the N-inv *type severe* group. Unfortunately, we could not obtain clear bands of total ribosomal S6 and phosphorylated ribosomal S6 in the rightmost lane. This result was obtained from mice in cohort 1. The blots were cropped, and the original blots are presented in Supplementary Fig. 6B with labels and Supplementary Fig. 7B without labels. (B) Hepatic ribosomal S6 phosphorylation levels in mouse models. The signal value of phosphorylated ribosomal S6 was normalized to the signal value of total ribosomal S6. In addition, the values were normalized to the average value of the SC group. Representative data are presented as mean ± standard deviation. *, $p < 0.05$ indicates statistical significance based on Dunnett's post-hoc test. SC, subcutaneous tumor model; N-inv, neural invasion model; phospho, phosphorylated; S6, ribosomal S6; p-S6, phosphorylated ribosomal S6.

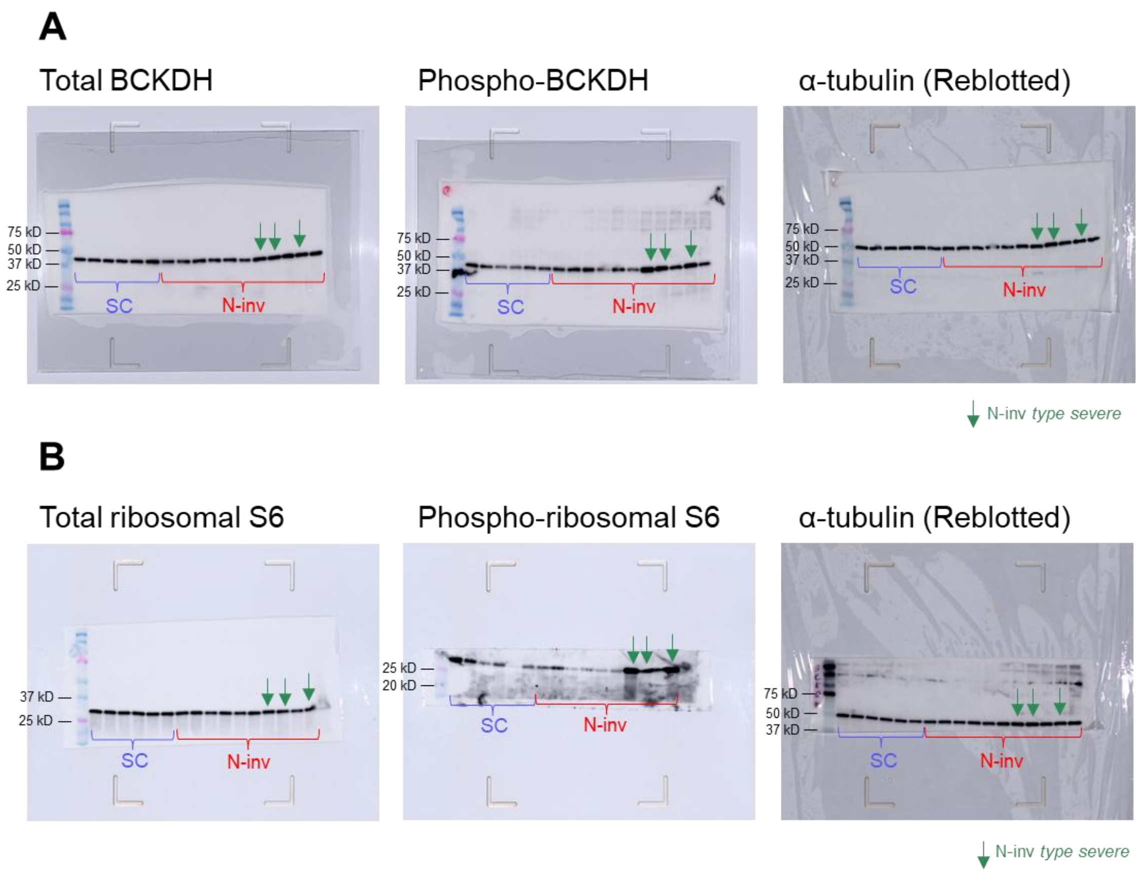

Supplementary Fig. 6. Original blots of the western blot experiments with labels. (A) Original blots of the cropped images shown in Fig. 2A. (B) Original blots of the cropped images shown in Supplementary Fig. 3A. Arrows indicate the bands for the N-inv *type severe* group. BCKDH, α-ketoisocaproate; phospho, phosphorylated.

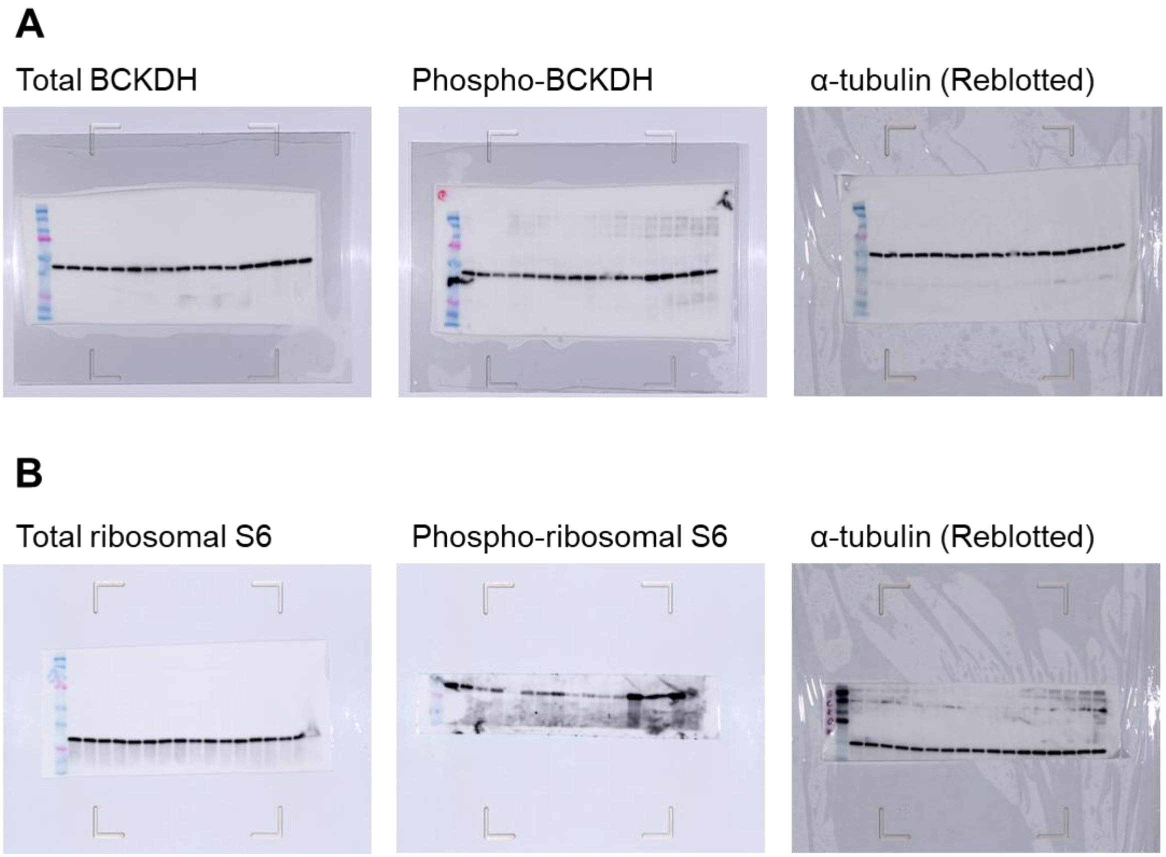

Supplementary Fig. 7. Original blots of the western blot experiments without any labels. (A) Original blots of the cropped images shown in Fig. 2A. (B) Original blots of the cropped images shown in Supplementary Fig. 3A. BCKDH, α-ketoisocaproate; phospho, phosphorylated.

Supplementary Table 1. Phenotypes of murine models in the animal cohort 1.

	SC-cont N = 7	SC N = 7	N-inv-cont N = 6-7	N-inv N = 9-11	*p*-value (SC vs N-inv)	*p*-value (N-inv-cont vs N-inv)
Weight (g)						
Whole body	22.9 ± 1.31	22.2 ± 1.05	23.0 ± 0.787	21.2 ± 0.959	0.114	*0.00306
Whole liver	1.00 ± 0.0520	0.931 ± 0.0928	1.02 ± 0.0621	0.824 ± 0.103	*0.0377	*<0.001
Epididymal fat	0.198 ± 0.0891	0.233 ± 0.130	0.237 ± 0.0869	0.0874 ± 0.0413	*0.00466	*0.00377
Kidney	0.209 ± 0.0100	0.192 ± 0.0215	0.205 ± 0.00882	0.197 ± 0.0239	(0.879)	(0.745)
Gastrocnemius muscle	0.156 ± 0.00966	0.150 ± 0.00883	0.149 ± 0.00842	0.149 ± 0.0128	(0.993)	(1.00)
Tumor	-	0.342 ± 0.420	-	0.211 ± 0.230	(0.44)	-
mRNA expression in spinal cord						
Gfap1	1.00 ± 0.146	1.06 ± 0.212	0.889 ± 0.147	1.68 ± 0.794	*0.0414	*0.00709

Only the right kidney and right gastrocnemius muscle were weighed because the human pancreatic cancer cells or PBS were injected into the left sciatic nerve or subcutaneous tissue of the left flanks of the mice. The epididymal fat was weighed bilaterally. mRNA expression values were adjusted using the ΔΔCT method, with cyclophilin as a reference gene. In addition, the values were normalized to the average value of SC-cont group. The mean ± standard deviation is shown. * $p< 0.05$, based on one-way ANOVA with Dunnett's post-hoc test. The *p*-values calculated based on Dunnett's post hoc test for comparisons that were not significant in the one-way ANOVA are shown in parentheses. SC, subcutaneous tumor model; N-inv, neural invasion model; SC-cont, sham control group for SC; N-inv-cont, sham control group for N-inv; Gfap1, glial fibrillary acidic protein 1.

Supplementary Table 2. Phenotypes of murine models in the cohort 2.

	SC<br>N = 10	N-inv<br>N = 12	p-value<br>(SC vs N-inv)
Weight (g)			
Whole body	23.1 ± 0.894	20.6 ± 1.49	*<0.001
Whole liver	0.928 ± 0.0810	0.755 ± 0.108	*<0.001
Epididymal fat	0.151 ± 0.0807	0.0513 ± 0.0222	*<0.001
Kidney	0.216 ± 0.0155	0.189 ± 0.0160	*<0.001
Gastrocnemius muscle	0.165 ± 0.0229	0.150 ± 0.0169	0.0864
Tumor	0.120 ± 0.0948	0.152 ± 0.112	0.486
mRNA expression in spinal cord			
Gfap1	1.00 ± 0.331	2.13 ± 0.973	*0.00229

Only the right kidney and right gastrocnemius muscle were weighed because the human pancreatic cancer cells or PBS were injected into the left sciatic nerve or subcutaneous tissue of the left flanks of the mice. The epididymal fat was weighed bilaterally. mRNA expression values were adjusted using the ΔΔCT method, with cyclophilin as a reference gene. In addition, the values were normalized to the average value of SC group. The mean ± standard deviation is shown. * $p < 0.05$, based on student's t-test. SC, subcutaneous tumor model; N-inv, neural invasion model; SC-cont, sham control group for SC; N-inv-cont, sham control group for N-inv; Gfap1, glial fibrillary acidic protein 1.

Supplementary Table 3. Demographic and clinical characteristics of patients with stage III pancreatic cancer.

		Low N-inv<br>N = 4	High N-inv<br>N = 14
Age (year)	Median (IQR)	60 (51/80)	65 (60/69)
Sex	Male	1	7
	Female	3	7
ECOG-PS	0	2	11
	1	2	3
Body weight (kg)	Median (IQR)	58.3 (55.5/60.0)	53.6 (47.8/61.0)
Weight loss in the past 6 months (%)	Median (IQR)	5.0 (3.3/5.9)	3.5 (-0.7/7.4)
Hemoglobin (g/dL)	Median (IQR)	11.2 (10.7/11.7)	12.5 (11.3/13.4)
Albumin (g/dL)	Median (IQR)	3.1 (3.0/3.2)	3.9 (3.6/4.2)
C-reactive protein (mg/dL)	Median (IQR)	0.13 (0.08/0.19)	0.19 (0.08/2.30)
Primary site	Head of the pancreas	4	14
	Body or tail of the pancreas	0	0
Ascites	Present	0	4
Biliary drainage	Present	3	4
UICC stage	III	4	14
CA19-9	Median (IQR)	1286 (766/5019)	163 (16/2678)
Regimen	Gemcitabine alone	3	10
	Gemcitabine plus S-1	0	1
	Gemcitabine plus investigational drug	0	1
	S-1	1	2

IQR, interquartile range; ECOG-PS, Eastern Cooperative Oncology Group performance status; UICC, Union for International Cancer control; CA19-9, carbohydrate antigen 19-9.

Supplementary Table 4. Circulating levels of BCAAs and BUN and changes in skeletal muscle mass according to the neural invasion status in patients with stage III pancreatic cancer.

Variables	Unit	Low N-inv<br>N = 4	High N-inv<br>N = 14	*p*-value
Plasma BCAA level at baseline	μM	251 ± 41.2	369 ± 66.2	**p* = 0.00447
Serum BUN level at baseline	μM	7.25 ± 1.26	14.0 ± 9.02	**p* = 0.00411
Change in skeletal muscle index over 3 months	cm^2/m^2	1.46 ± 5.19	−9.77 ± 9.02	**p* = 0.0334

Based on the computed tomography images, low N-inv was defined as perivascular soft tissue (PVST) intact to neither the superior mesenteric artery (SMA) nor the celiac artery (CeA). High N-inv was defined as the PVST in contact with the SMA or CeA. The mean ± standard deviation is shown. * $p < 0.05$, based on Student's t-test. BCAA, branched-chain amino acid; BUN, blood urea nitrogen; N-inv, neural invasion.

Supplementary Table 5. Demographic and clinical characteristics of patients with stage IV pancreatic cancer.

		N (%)
Age (year)	Median (IQR)	66 (63/74)
Sex	Male	29 (61.7)
	Female	18 (38.3)
ECOG-PS	0	24 (51.1)
	1	18 (38.3)
	2	4 (8.6)
	3	1 (2.1)
Body weight (kg)	Median (IQR)	56.0 (48.4/65.7)
Weight loss in the past 6 months (%)	Median (IQR)	6.2 (3.3/12.2)
Hemoglobin (g/dL)	Median (IQR)	14.0 (11.5/14.0)
Albumin (g/dL)	Median (IQR)	3.9 (3.5/4.2)
C-reactive protein (mg/dL)	Median (IQR)	0.7 (0.3/1.8)
Primary site	Head of the pancreas	11 (23.4)
	Body or tail of the pancreas	36 (76.6)
Ascites	Present	23 (48.9)
Biliary drainage	Present	4 (8.5)
UICC stage	IV	66 (80.5)
CA19-9	Median (IQR)	3268 (278/11619)
Regimen	Gemcitabine alone	11 (23.4)
	Gemcitabine doublets	18 (38.3)
	FOLFIRINOX	11 (13.3)
	Best supportive care	7 (14.9)

IQR, interquartile range; ECOG-PS, Eastern Cooperative Oncology Group performance status; UICC, Union for International Cancer control; CA19-9, carbohydrate antigen 19-9; nab-PTX, nanoparticle albumin-bound paclitaxel; FOLFIRINOX, 5-fluorouracil/leucovorin combined with irinotecan and oxaliplatin.

Supplementary Table 6. Parameters evaluated in cohorts 1 and 2.

	Animal cohort 1<br>SC-cont (N = 7), SC (N = 7), N-inv-cont (N = 7) and N-inv (N = 11)	Animal cohort 2<br>SC (N = 10) and N-inv (N = 12)
Body weight change	✓	✓
Cumulative food intake	✓	-
Weight		
Whole body	✓	✓
Whole liver	✓	✓
Epididymal fat	✓	✓
Kidney	✓	✓
Gastrocnemius muscle	✓	✓
Tumor	✓	✓
CSA of the muscle fibers	-	✓
Whole body leucine flux	-	✓
BCKA in the liver	✓	-
Plasma metabolite		
NEFA	✓	✓
Ketone	-	✓
Glucose	-	✓
BUN	✓	✓
Amino acids	✓	✓
mRNA expression		
Spinal cord *Gfap1*	✓	✓
Gastrocnemius muscle *Murf1*	✓	✓
Liver *Hmgcs2*	✓	✓
Gastrocnemius muscle *Bcat2*	✓	-
Epididymal fat *Bcat2*	✓	-
Liver *Bcat2*	✓	-
Western blot		
Liver total BCKDH	✓	-
Liver P-BCKDH	✓	-
Liver ribosomal S6	✓	-
Liver ribosomal P-S6	✓	-

✓, evaluated and -, not evaluated. *Bcat2* mRNA expression in tissue and BCKA and phosphorylation levels of BCKDH and ribosomal S6 in the liver were not evaluated in cohort 2, because infused leucine could affect these parameters. SC, subcutaneous tumor model; N-inv, neural invasion model; SC-cont, sham control group for SC; N-inv-cont, sham control group for N-inv; BCKA, branched-chain keto acids; *Gfap1*, glial fibrillary acidic protein 1; *Murf1*, mRNA expression level of muscle ring finger protein 1; *Hmgcs2*, mRNA expression level of 3-hydroxy-3-methylglutaryl-CoA synthase 2; *Bcat2*, mRNA levels of branched chain amino acid transaminase 2; CSA of the muscle fibers, cross-sectional area of the gastrocnemius muscle fibers; NEFA, non-esterified fatty acid; BUN, blood urea nitrogen; BCKDH, branched-chain α-keto acid dehydrogenase; p-BCKDH, phosphorylated BCKDH; S6, ribosomal S6; p-S6; phosphorylated ribosomal S6.

Supplementary Table 7. Primers used in this study.

Mouse *Cyclophilin*	Forward	5′-TGGAAGAGCACCAAGACAGACA-3′
	Reverse	5′-TGCCGGAGTCGACAATGAT-3′
Mouse *Gfap1*	Forward	5′-ACCAGCTTACGGCCAACAGTG-3′
	Reverse	5′-TGTCTATACGCAGCCAGGTTGTTC-3′
Mouse *Hmgcs2*	Forward	5′-TGCTATGCAGCCTACCGCAAGA-3′
	Reverse	5′-GCCAGGGATTTCTGGACCATCT-3′
Mouse *Murf1*	Forward	5′-GAGGGCCATTGACTTTGGGA-3′
	Reverse	5′-TTTACCCTCTGTGGTCACGC-3′
Mouse *Bcat2*	Forward	5′-AGCTCATCCTGCGCTTCCA-3′
	Reverse	5′-GCACGTCTGCAGATCCAACC-3′

Gfap1, glial fibrillary acidic protein 1; *Hmgcs2*, 3-hydroxy-3-methylglutaryl-CoA synthase 2; *Murf1*, muscle ring finger protein 1; *Bcat2*, branched chain amino acid transaminase 2.
